# Safe and effective hybrid endoscopic submucosal dissection with ALL IN ONE snare in porcine gastric model (with video)

**DOI:** 10.1038/s41598-024-61031-4

**Published:** 2024-05-02

**Authors:** Lang Yang, Xian-zong Ma, Hui Su, Jie Zhang, Jian-qiu Sheng, Peng Jin

**Affiliations:** 1 Senior Department of Gastroenterology, The First Medical Center of Chinese, PLA General Hospital, Beijing, 100853 China; 2 Department of Gastroenterology, The Seventh Medical Center of Chinese, PLA General Hospital, No. 5 Nan Men Cang, Dong Cheng District, Beijing, 100700 China; 3 Medical School of Chinese PLA, Beijing, 100853 China

**Keywords:** Hybrid endoscopic submucosal dissection, Snare, Porcine model, Procedure time, Stomach, Gastroenterology, Colonoscopy

## Abstract

This study aimed to evaluate the safety and efficiency of hybrid endoscopic submucosal dissection (H-ESD) using a newly developed ALL IN ONE (AIO) snare. This was a matched control study in a porcine model. Five paired simulated stomach lesions 2–2.5 cm in size were removed by H-ESD using an AIO snare or conventional ESD (C-ESD) using an endoscopic knife. The outcomes of the two procedures were compared, including en-bloc resection rates, procedure times, intraprocedural bleeding volumes, muscular injuries, perforations, thicknesses of the submucosal layer in resected specimens, and stomach defects. All simulated lesions were resected en-bloc. Specimens resected by H-ESD and C-ESD were similar in size (7.68 ± 2.92 vs. 8.42 ± 2.42 cm^2^; *P* = 0.676). H-ESD required a significantly shorter procedure time (13.39 ± 3.78 vs. 25.99 ± 4.52 min; *P* = 0.031) and submucosal dissection time (3.99 ± 1.73 vs. 13.1 ± 4.58 min; *P* = 0.003) versus C-ESD; H-ESD also yielded a faster dissection speed (241.37 ± 156.84 vs. 68.56 ± 28.53 mm^2^/min; *P* = 0.042) and caused fewer intraprocedural bleeding events (0.40 ± 0.55 vs. 3.40 ± 1.95 times/per lesion;* P* = 0.016) than C-ESD. The thicknesses of the submucosal layer of the resected specimen (1190.98 ± 134.07 vs. 1055.90 ± 151.76 μm; *P* = 0.174) and the residual submucosal layer of the stomach defect (1607.94 ± 1026.74 vs. 985.98 ± 445.58 μm; *P* = 0.249) were similar with both procedures. The AIO snare is a safe and effective device for H-ESD and improves the treatment outcomes of gastric lesions by shortening the procedure time.

## Introduction

Endoscopic submucosal dissection (ESD) is a microinvasive technique for the en-bloc removal of precancerous lesions or early-stage gastrointestinal cancer with limited lymph node metastasis risk^1^. Compared with endoscopic mucosal resection (EMR), ESD achieves a high en-bloc resection rate but increases the risk of perforation and bleeding^2^. Moreover, ESD requires a longer learning curve due to technique complexity^3^. The standard ESD protocol consists of the following steps: lesion marking, submucosal injection, mucosal incision, submucosal dissection, and hemostasis using electrical equipment or devices, such as needles, knives, and hemostatic forceps^1^.

**Figure 1:**
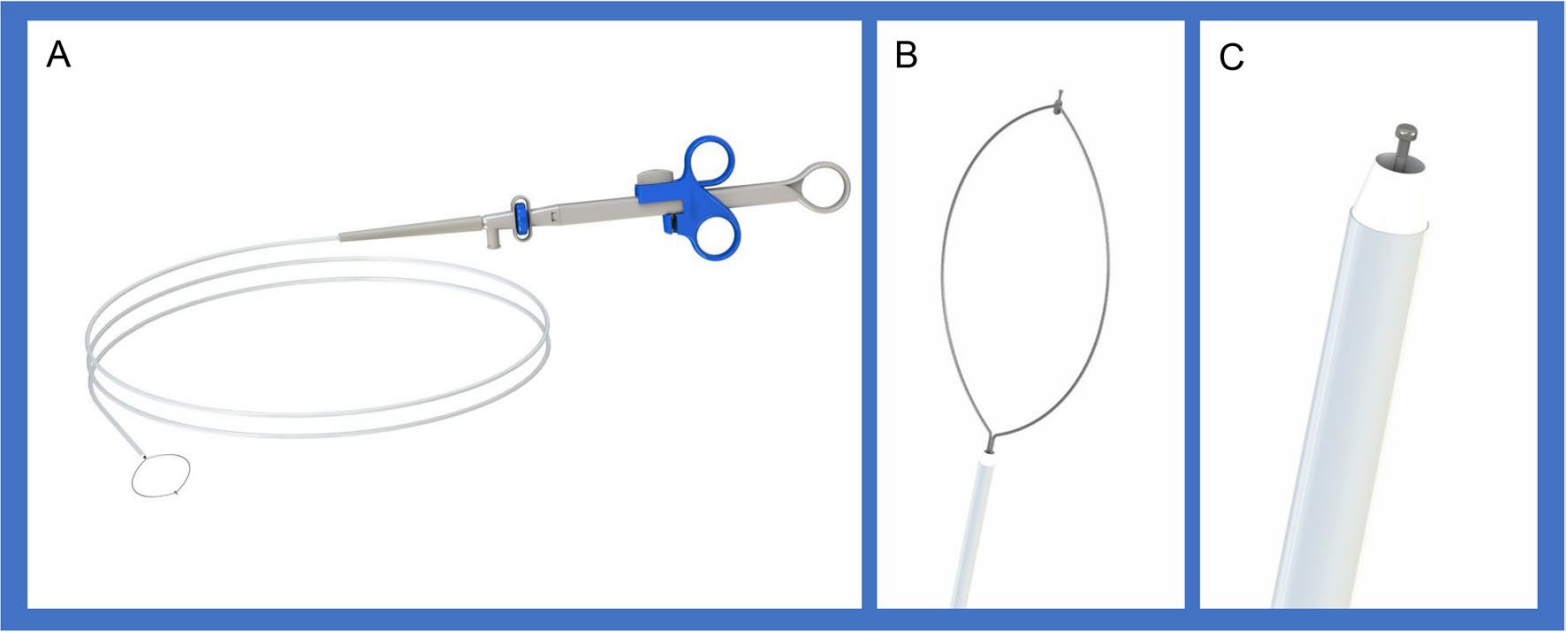
Picture of ALL IN ONE snare. (**A**) Overview of the snare. (**B**) The tip was in front of the snare. (**C**) A needle-knife with a knob-shaped tip can achieve precutting and dissection.

Hybrid ESD (H-ESD) was developed to simplify the en-bloc resection of selected lesions. Briefly, after mucosal incision and partial submucosal dissection, the lesion is removed using a snare^4^. H-ESD has been demonstrated safe and effective for the resection of colorectal lesions, with a shorter procedure duration, fewer complications, and no difference in recurrence versus conventional ESD (C-ESD)^5^. New devices with multiple functions, including marking, incision, dissection, and hemostasis, have been developed to increase the efficiency and lower the cost of H-ESD, such as the SOUTEN snare (ST1850-20, Kaneka, Medix, Tokyo, Japan)^6^ and Flat Adenoma Resection Instruments (FARIn; Endox-Feinmechanik GmbH, Bad Urach, Germany)^7^. Recently, a novel multifunctional snare [ALL IN ONE (AIO); LeoMed, Changzhou, China] was introduced for efficient H-ESD (Fig. 1). AIO performs integrated injection and argon plasma coagulation (APC) functions, enabling selective initial range marking, submucosal injection, mucosal incision and dissection, and hemostasis to ensure complete, independent, and safe ESD operation^8,9^. In this study, we investigated the safety and efficiency of the AIO snare for H-ESD in a porcine model.

## Methods

### Study design

This was a matched-control study to evaluate the safety and efficiency of the AIO snare for H-ESD versus a traditional endoscopic knife for C-ESD in a porcine model in vivo. The study was conducted at the experimental animal center of Pengli Testing Technology (Shanghai, China) Co., Ltd. (Shanghai, China) in accordance with Animal Research Reporting In *vivo* Experiments guidelines^10^. This study was approved by the Institutional Review Board of Pengli Testing Technology (Shanghai, China) Co., Ltd. Animal experiments were conducted according to guidelines for the ethical review of laboratory animal welfare (People’s Republic of China National Standard GB/T 35892-2018) and the institutional guidelines.

### Animals, devices, and procedure

Two matched healthy white pigs with pre-procedure weights of 35–40 kg were used. The animals underwent fasting, general anesthesia with endotracheal intubation, and monitoring. The experiment was conducted using a GIF-Q260J (Olympus, Japan) endoscope with a transparent cap at the tip. A high-frequency generator (VIO 200 S, ERBE, Germany) and water jet (Olympus, Japan) were used. A total of ten (matched 2 × 5) simulated lesions 2–2.5 cm in length were marked using the transparent cap as reference (3 in the stomach body and 2 in the antrum at similar locations in each pig. and dissected by 3 endoscopists (Dr. Peng Jin, and Dr. Lang Yang, and Dr. Hui Su), all the endoscopists were trained but with a little experience of needle-type knife (MK-T-2-195) and AIO snare (less than 5 cases for each). For C-ESD, a traditional endoscopic needle-type knife (MK-T-2-195, Micro-Tech, Nanjing, China) was used for marking, mucosal incision, and dissection. A 3 cm AIO snare with a 2 mm tip was used for H-ESD. In addition, submucosal injection with saline plus indigo was conducted by using a disposable needle (M00518351, Boston Scientific, USA) in both H-ESD and C-ESD group to decrease bias. In H-ESD, the lesion was marked by AIO snare, followed by submucosal injection by using disposable needle. Then, circumferential mucosal incision deep into the submucosal layer was made by protruded tip of AIO snare, the lesion was removed appropriately using the AIO snare after partial submucosal dissection depending on experience (Supplementary Video). Intraprocedural bleeding was controlled by knife/AIO snare or hemostatic forceps (HBF-23/2000, Micro-Tech, Nanjing, China), depending on the amount of bleeding. The specimens were fixed and cut for histological analysis.

### Outcome measures and definitions

The primary outcome was procedure time, defined as the time from the start of the mucosal incision to the completion of lesion resection, including submucosal injection, mucosal incision, dissection/snaring, and homeostasis in each procedure. The time of submucosal dissection was measured from the beginning to the end of submucosal dissection, including dissection using a knife or snare and the time required for additional submucosal injection and homeostasis.

The secondary outcomes were as follows: en-bloc resection rates, submucosal resection speeds, adverse events (intraprocedural bleeding, perforation, and muscular injury), thicknesses of the submucosal layer in the resected specimen, and stomach defects. Intraprocedural bleeding was defined as bleeding during submucosal dissection, and the number of intraoperative bleedings, hemostasis using hemostatic forceps were recorded. Perforation was observed and recorded during the experimental procedure. Muscular injury was defined as destroyed muscle bundle observed under endoscopy or degeneration or necrosis of muscle cells in hematoxylin–eosin-stained slides. Dissection speed was calculated as the specimen size divided by the time of submucosal dissection. The depth of the submucosal layer of resected specimens and stomach defects was measured as previously described^11^. Briefly, the depth of the submucosal layer was calculated as the area of the submucosal layer divided by the corresponding muscular length^11^. In addition, Dr. PJ and LY had experience of more than 150 gastric ESD cases were classified as senior endoscopists, and Dr. HS had experience of less than 50 gastric ESD cases was classified as junior endoscopist.

### Sample size calculation

According to a preliminary experiment, the mean C-ESD procedure time is approximately 35 min with a standard deviation (SD) of 5 min, while that for H-ESD is 18 min for resecting lesions 2–3 cm in size. We used PASS 11.0 software for sample size estimation. With a power of 0.90 and two sides alpha of 0.05, the sample size should be 4 in each group. To compensate for a 20% dropout rate, we aimed to include 5 lesions in each group.

### Statistical analysis

Categorical data are represented by the number of cases and percentage, and comparisons between the 2 procedures were performed by the chi-square or Fisher’s exact tests. Normally distributed quantitative data are shown as the mean and SD and were analyzed using Student’s *t* test. The generalized linear model was used to analyze the factors influencing procedure time. *P* values < 0.05 were considered to indicate significance. All statistical analyses were performed using SPSS 26.0 software (IBM Corporation, Armonk, NY).

### Ethics approval and consent to participate

This study was approved by the Institutional Review Board of Pengli Testing Technology (Shanghai, China) Co., Ltd.

## Results

**Figure 2:**
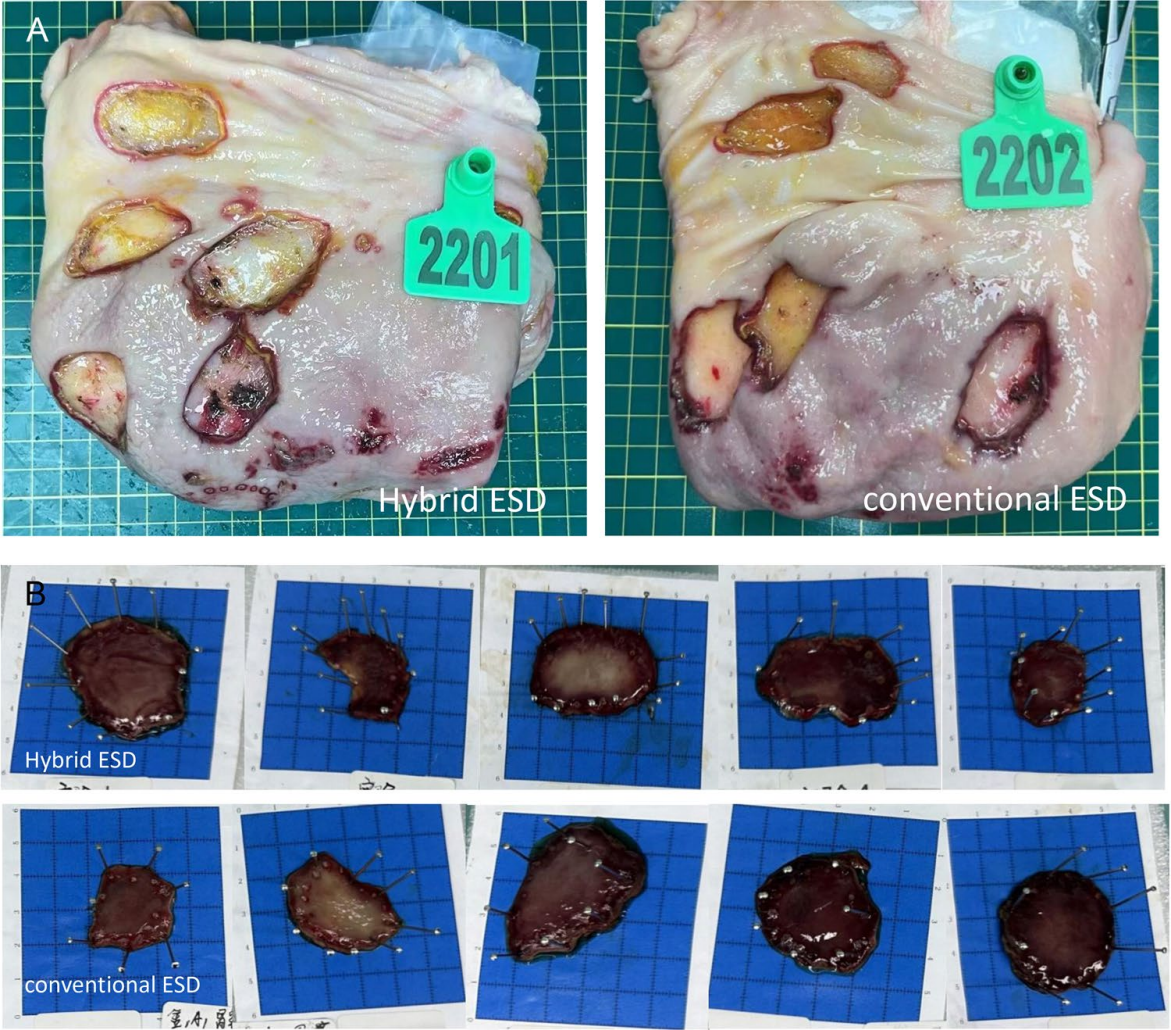
Resected specimens and stomach defects in the hybrid and conventional ESD groups. (**A**) Stomach defects showed a smooth wound surface, and no perforations occurred; (**B**) the lesions were subjected to en-bloc resection.

**Figure 3:**
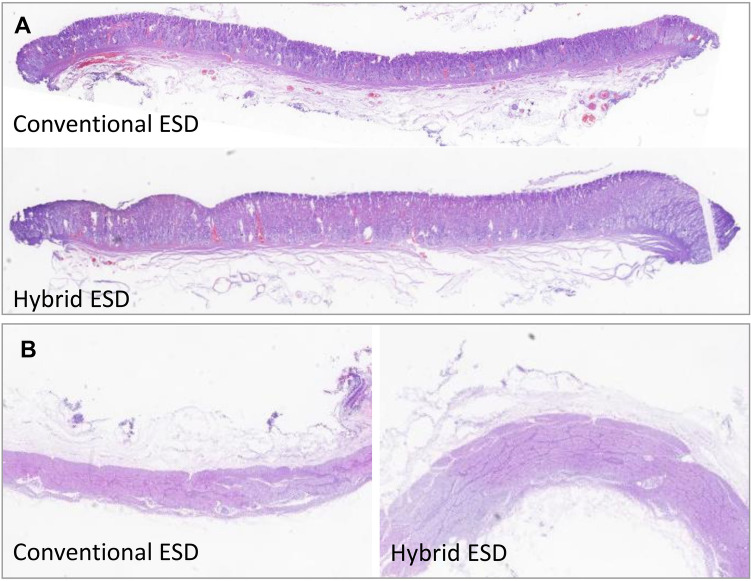
Histology of the resected specimens and stomach defects. (**A**) Histology of resected specimens showed intact muscularis mucosae in hybrid and conventional ESD. (**B**) The stomach defect showed intact muscularis propria in both groups.

All lesions were resected en-bloc, and no perforations occurred (Fig. 2). Specimens resected by H-ESD and C-ESD were similar (7.68 ± 2.92 vs. 8.42 ± 2.42 cm^2^; *P* = 0.676). The H-ESD procedure time was significantly shorter than that for C-ESD (13.39 ± 3.78 vs. 25.99 ± 4.52 min;* P* = 0.031), as was the submucosal dissection time (3.99 ± 1.73 vs. 13.1 ± 4.58 min; *P* = 0.003), but similar mucosal incision time was observed in H-ESD and C-ESD (9.48 ± 5.14 vs. 12.88 ± 6.64 min; *P* = 0.382); a faster dissection speed was also obtained using H-ESD (241.37 ± 156.84 vs. 68.56 ± 28.53 mm^2^/min; *P* = 0.042). No perforation occurred; however, H-ESD resulted in fewer number of intraprocedural bleeding than C-ESD (0.40 ± 0.55 vs. 3.40 ± 1.95; *P* = 0.016), but similar number of hemostasis events using hemostatic forceps (0.20 ± 0.45 vs. 0.40 ± 0.89; P = 0.267). Two tiny muscular injuries were observed under endoscopy in C-ESD group, but there is no statistic difference between the two procedures (*P* = 0.444). In addition, the histological analysis of the resected specimens showed intact muscularis mucosae and muscularis propria without degeneration and necrosis corresponding to stomach defects in hybrid and conventional ESD. Moreover, the thickness of submucosal layer of the resected specimen was similar in H-ESD and C-ESD (1190.98 ± 134.07 vs. 1055.90 ± 151.76 μm; *P* = 0.174), as was the thickness of the residual submucosal layer of the stomach defect (1607.94 ± 1026.74 vs. 985.98 ± 445.58 μm; *P* = 0.249) (Table 1 and Fig. 3).

**Table 1:** Comparison of treatment outcomes of endoscopic submucosal dissection between two groups.

Characteristic	Hybrid ESD (N = 5)	Conventional ESD (N = 5)	P value
Location			
Antrum of stomach	2	2	0.738
Gastric body	3	3	
Procedure time (mean ± SD, min)	13.39 ± 3.78	25.99 ± 4.52	0.031
Time of mucosal incision (mean ± SD, min)	9.48 ± 5.14	12.88 ± 6.64	0.382
Time of submucosal dissection (mean ± SD, min)	3.99 ± 1.73	13.11 ± 4.58	0.003
En-bloc resection	5	5	
Perforation	0	0	
Intraprocedural bleeding (mean ± SD, No.)	0.40 ± 0.55	3.40 ± 1.95	0.016
Hemostasis events using hemostatic forceps (mean ± SD, No.)	0.20 ± 0.45	0.40 ± 0.89	0.267
Specimen size (mean ± SD, cm^2^)	7.68 ± 2.92	8.42 ± 2.42	0.676
Dissection speed (mean ± SD, mm^2^/min)	241.37 ± 156.84	68.56 ± 28.53	0.042
Muscular injury	0	2	0.444*
Depth of Submucosal layer of resected specimen (mean ± SD, μm)	1190.98 ± 134.07	1055.90 ± 151.76	0.174
Thickness of residual submucosal layer on defects (mean ± SD, μm)	1607.94 ± 1026.74	985.98 ± 445.58	0.249

*The P value was analyzed by Fisher’s exact test. Number, No.

A generalized linear regression model was used to analyze the effects of AIO, operator, location, intraprocedural bleeding, and specimen size on procedure time. The results show that AIO is an independent factor affecting procedure time, and the use of AIO can significantly reduce the procedure time (*P* = 0.004) (Table 2).

**Table 2:** Generalized linear regression model analysis of procedure time.

Variables	B	S.E	X^2^	P value
ESD				
H-ESD (AIO)	− 12.600	4.320	8.508	0.004
C-ESD (reference)				
Operator				
Junior endoscopist	6.810	7.023	0.940	0.332
Senior endoscopist (reference)				
Location				
Gastric body	7.228	4.545	1.699	0.192
Gastric antrum (reference)				
Intraprocedural bleeding				
Yes	5.712	6.152	0.862	0.353
No (reference)				
Specimen size	1.290	1.139	1.282	0.258

## Discussion

This study evaluated the safety and efficiency of the AIO snare for H-ESD in the resection of gastric lesions in a live porcine model. In these simulated gastric lesions, the AIO snare yielded a comparable en-bloc resection rate to that achieved with the traditional endoscopic knife but required a shorter procedure time.

Endoscopic resection is a less invasive treatment for early gastric cancer with limited lymph node metastasis or precancerous lesions^2^. EMR and polypectomy were initially introduced for early-stage gastric neoplasms, which are safe and effective. However, conventional gastric EMR for larger lesions often results in an insufficient resection margin due to snare slippage, potentially leading to piecemeal resection, which complicates the pathological evaluation of specimens and is associated with a high local recurrence rate^12,13^. To overcome the technique limitation of EMR, ESD adopts a mucosal lesion incision followed by submucosal dissection instead of a snare, theoretically allowing preplanned en-bloc resection of target lesions of any size. ESD is significantly superior to EMR in achieving higher curative resection rates and lower recurrence rates^14^. Thus, the European guidelines recommend ESD as the first-line endoscopic treatment for gastric superficial lesions with null/very low lymph node metastasis risk and EMR as an alternative for elevated lesions < 10 mm with a low likelihood of advanced histology^15^. However, ESD requires a longer procedure time and is associated with a higher rate of adverse events since it is more technically difficult than EMR and requires more specialized skills.

H-ESD is an alternative method for endoscopic resection that bridges the gap between ESD and EMR. H-ESD involves mucosal incision, partial submucosal dissection of the lesion, and snare resection. H-ESD was initially used as a rescue method for resecting colorectal lesions and later for the planned resection of target lesions^16^. In H-ESD, a mucosal incision is made by an endoscopic knife or SOUTHEN snare^17^. A systemic analysis showed that H-ESD is safe and effective for removing colorectal lesions, with a shorter procedure duration, fewer adverse events, and no difference in recurrence rates compared to C-ESD; however, H-ESD is associated with a lower en-bloc resection rate^5^. ESD is the best endoscopic modality for achieving en-bloc resection for lesions larger than 2 cm. However, regarding colorectal lesions, those 2–3 cm in size were found to be most suitable for H-ESD^18^. H-ESD has also been used for esophageal lipoma^19^, anal canal fibroma^20^, rectal neuroendocrine tumor^21,22^ resection, and even for full-thickness resection of T2 colorectal cancer^23^.

H-ESD effectively reduces the procedure time for gastric dysplastic lesions. A randomized trial showed that H-ESD with SOUTHEN requires a significantly shorter procedure time, with high and comparable curability rates and safety profiles to those of C-ESD for differentiated intramucosal lesions without ulceration < 20 mm in diameter^6^. Similarly, Chiyo et al. reported that cost-efficient H-ESD using SOUTEN is acceptable for resecting gastric neoplasms < 15 mm^17^. In the current study, we tested the newly developed, multifunctional AIO snare, which offers the following advantages: (1) the conical head allows the protective tube head end to be easily inserted into the tissue opening, and the linker between the tip and snare are embedded designed for the tube, which stabilize the length of protruded tip for subsequent mucosal incision and submucosal dissections, we found that the AIO snare achieved the similar mucosal incision time than traditional needle-type knife. Those suggest that the cut or dissection ability of AIO snare may not inferior to traditional needle type knife; (2) the knob on the handle ensures that the knife head is positioned accurately and does not move when the hand is moved by mistake, ensuring a stable operational process; and (3) the AIO is equipped with argon gas and water injection ports that can selectively inject low viscoelasticity liquid such as water or saline and APC, and perform injections, electroincisions, electrocoagulation, and edge electroincision and electrocoagulation to achieve selective initial range marking, submucosal injection, mucosal incision and stripping, and hemostasis functions, but the function of APC and injection was not involved in test to decrease bias. In the current study, the AIO snare was tested for H-ESD for lesions 2–2.5 cm in size, which is larger than previous reports^6,17^, and en-bloc resection was achieved for all lesions. In addition, H-ESD required a shorter procedure time and achieved a faster dissection speed than C-ESD, indicating that H-ESD can be expanded to include larger lesions and achieve en-bloc resection. During H-ESD, the mucosal incision outside the marking dots and the submucosa is partially dissected, enabling subsequent snaring to easily achieve en-bloc resection. In addition, to prevent muscular injury, it is better to elevate the lesion by adequate submucosal injection and/or lift the snare away from the muscular propria when the snare is cut. In current study, two tiny muscular injuries were observed under endoscopy in C-ESD, but not in pathological slides, possible explanation was that the muscular injuries were too tiny and missed by pathological sampling. Moreover, fewer intraprocedural bleeding events occurred with H-ESD than with C-ESD, which may be one reason that H-ESD simplified the dissection process. Additionally, the resection depths with H-ESD and C-ESD were similar (mean 1190 μm). However, the H-ESD resection depth in the human population requires further investigation. In practice, the dissection depth can adjust and cut just above the muscle layer in C-ESD, whereas, the adjusting the dissection depth is challenging when using snare resection in H-ESD. Thus, carefully pre-resection evaluation and selection of mucosal lesions is essential for successful planned H-ESD. In addition, in case of submucosal invasive lesion suspected, the step of snare resection in H-ESD should be abandoned and transit to conventional dissection by protruded tip of AIO snare.

The strengths of this study are as follows. (1) This was a matched-control study to test the safety and efficiency of a newly developed AIO snare for H-ESD, and this snare was demonstrated to safely achieve en-bloc resection with a shorter procedure time than that of C-ESD using an endoscopic knife. (2) The experiment was conducted by three endoscopists with different experience levels, indicating that the AIO snare is easy to use, even for less experienced endoscopists. (3) H-ESD was achieved using one device (the AIO snare), eliminating the need for device exchange during the procedure and potentially reducing ESD durations and costs. However, this study had some limitations. First, this preliminary study was conducted in a porcine model with small number simulated lesions without the ulceration associated with increased submucosal fibrosis resulting from invasive cancer or pretreatment biopsy. Therefore, the results need to be validated in a population study. In addition, the area of partial submucosal dissection in H-ESD was determined by endoscopists’ experience, which may influence the snare resection difficulties and procedure time. In Bae JH et al.’s study on H-ESD for colorectal lesions, the snared submucosal tissue was resected when the maximum length of the slit on the handle was < 5 mm^24^. However, how to predict the area of undissected submucosal tissue for safe en-bloc resection in gastric neoplasia remains to be illustrated.

## Conclusion

The AIO snare was found to be a safe and effective device for H-ESD, with a shorter procedure time and similar en-bloc resection rate for gastric lesions to that achieved with C-ESD. Those results providing guidance for the next stage of clinical trials.

### Supplementary Information

Supplementary Video 1.

## Data Availability

All materials are commercially available, and the datasets used and/or analyzed during the current study are available from the corresponding author on reasonable request.

## References

[1] Esaki M, Ihara E, Gotoda T (2021). Endoscopic instruments and techniques in endoscopic submucosal dissection for early gastric cancer. Expert. Rev. Gastroenterol. Hepatol..

[2] Vasconcelos AC, Dinis-Ribeiro M, Libânio D (2023). Endoscopic resection of early gastric cancer and pre-malignant gastric lesions. Cancers (Basel).

[3] Yoshida M, Kakushima N, Mori K, Igarashi K, Kawata N, Tanaka M (2017). Learning curve and clinical outcome of gastric endoscopic submucosal dissection performed by trainee operators. Surg. Endosc..

[4] Okamoto Y, Oka S, Tanaka S, Nagata S, Kunihiro M, Kuwai T (2022). Indications and outcomes of colorectal hybrid endoscopic submucosal dissection: A large multicenter 10-year study. Surg. Endosc..

[5] McCarty TR, Bazarbashi AN, Thompson CC, Aihara H (2021). Hybrid endoscopic submucosal dissection (ESD) compared with conventional ESD for colorectal lesions: A systematic review and meta-analysis. Endoscopy.

[6] Esaki M, Ihara E, Sumida Y, Fujii H, Takahashi S, Haraguchi K (2023). Hybrid and conventional endoscopic submucosal dissection for early gastric neoplasms: A multi-center randomized controlled trial. Clin. Gastroenterol. Hepatol..

[7] Gölder SK, Schaller T, Farin G, Messmann H, Probst A (2016). Partially insulated cutting instruments for hybrid endoscopic submucosal dissection—the Flat Adenoma Resection Instruments (FARIn). Endoscopy.

[8] Kou, P., Ge, Q., & Zhang, Z. The utility model relates to a tool head assembly of a medical electric knife and a medical electric knife. CHINA, 202222751354[P], 2023–10–20. https://pss-system.cponline.cnipa.gov.cn/retrieveList?prevPageTit=changgui.

[9] Zhou, P., Cai, M., Ge, Q. *et al.* The utility model relates to a tool head assembly of a new medical electric knife and a new medical electric knife. CHINA, CN202111337053.9 [P]. 2022–08–05. https://pss-system.cponline.cnipa.gov.cn/documents/detail?prevPageTit=changgui.

[10] PercieduSert N, Hurst V, Ahluwalia A, Alam S, Avey MT, Baker M (2020). The ARRIVE guidelines 2.0: Updated guidelines for reporting animal research. PLoS Biol..

[11] Ito A, Suga T, Ota H, Tateiwa N, Matsumoto A, Tanaka E (2018). Resection depth and layer of cold snare polypectomy versus endoscopic mucosal resection. J. Gastroenterol..

[12] Ono H, Kondo H, Gotoda T, Shirao K, Yamaguchi H, Saito D (2001). Endoscopic mucosal resection for treatment of early gastric cancer. Gut.

[13] Horiki N, Omata F, Uemura M, Suzuki S, Ishii N, Fukuda K (2012). Risk for local recurrence of early gastric cancer treated with piecemeal endoscopic mucosal resection during a 10-year follow-up period. Surg. Endosc..

[14] Ono H, Yao K, Fujishiro M, Oda I, Uedo N, Nimura S (2021). Guidelines for endoscopic submucosal dissection and endoscopic mucosal resection for early gastric cancer (second). Dig. Endosc..

[15] Pimentel-Nunes P, Libânio D, Bastiaansen B, Bhandari P, Bisschops R, Bourke MJ (2022). Endoscopic submucosal dissection for superficial gastrointestinal lesions: European Society of Gastrointestinal Endoscopy (ESGE) Guideline - Update 2022. Endoscopy.

[16] Gostout CJ, Knipschield MA (2012). Submucosal endoscopy with mucosal resection: A hybrid endoscopic submucosal dissection in the porcine rectum and distal colon. Gastrointest. Endosc..

[17] Chiyo T, Kobara H, Nishiyama N, Nakatani K, Tada N, Koduka K (2022). Acceptability of hybrid endoscopic submucosal dissection using multifunctional snare for small-sized gastric neoplasms: A prospective observational study. J. Gastrointest. Liver Dis..

[18] Gorospe EC, Wong Kee Song LM (2016). Hybrid endoscopic submucosal dissection in the colon: Cutting corners or trimming fat. Gastrointest. Endosc..

[19] Deshmukh A, Elmeligui A, Parsa N, Tejedor-Tejada J, Nieto J (2021). Successful removal of a giant esophageal lipoma with hybrid endoscopic submucosal dissection. VideoGIE.

[20] Okamoto T, Ikeya T, Fukuda K (2022). Hybrid endoscopic submucosal dissection for anal canal fibroma. VideoGIE.

[21] Nasu T, Esaki M, Shoguchi Y, Bai X, Minoda Y, Ogino H (2022). Traction-assisted hybrid endoscopic submucosal dissection for small rectal neuroendocrine tumors. Endoscopy.

[22] Gravito-Soares M, Gravito-Soares E, Amaro P, Cunha I, Fraga J, Tomé L (2019). Endoscopic resection of a rectal neuroendocrine tumor: Hybrid endoscopic submucosal dissection. GE Port. J. Gastroenterol..

[23] Wilson N, Abdallah M, Bilal M (2023). Hybrid endoscopic submucosal dissection and endoscopic full-thickness resection for complete resection of a T2 colorectal adenocarcinoma in a nonsurgical candidate. Gastrointest. Endosc..

[24] Bae JH, Yang DH, Lee S, Soh JS, Lee S, Lee HS (2016). Optimized hybrid endoscopic submucosal dissection for colorectal tumors: A randomized controlled trial. Gastrointest. Endosc..

